# Risk, Obligation, and Public Noncompliance with Mobility Directives in China during the COVID-19 Pandemic

**DOI:** 10.3390/ijerph191811505

**Published:** 2022-09-13

**Authors:** Chunhui Zheng, Jia Zhang, Lili Qian, Yuling Zhang

**Affiliations:** 1 School of Management, Guangzhou University, Guangzhou 510006, China; 2 International School of Cultural Tourism, Zhejiang University City College, Hangzhou 310015, China; 3 Department of Tourism, Foshan University, Guangzhou 528051, China

**Keywords:** risk-related constraint, travel motivation, negotiation, maladaptive response, perceived obligation, COVID-19

## Abstract

Human mobility greatly increases the risk of epidemic transmission. This study examines the psychological mechanism of individuals’ noncompliance with public health directives and their choice to travel amidst threats through two rounds of surveys (N = 1473 in total) in China at different stages of the COVID-19 pandemic. This research revealed the relative strength of the motivating and impeding factors that determined behavioral intention. In subtle internal conflicts, maladaptive responses (e.g., wishful thinking, denial, fatalism) were identified as a significant factor in negotiating risk-related constraints and encouraging risky travel behavior. Interestingly, both those who traveled amidst threats and those who did not travel agreed that they had social obligations for epidemic prevention. The results demonstrated that obligation could have an indirect negative impact on behavioral intention only via attitude. By unveiling the psychological mechanism of individuals’ noncompliance with health directives and travel during the pandemic, this study can aid in the development of appropriate operational strategies to manage population mobility during crises.

## 1. Introduction

The global outbreak of a novel coronavirus in late 2019 has resulted in tremendous economic, emotional, and mental costs [1,2]. The unprecedented COVID-19 pandemic swept the world quickly, while population mobility and travel further accelerated the transmission of the virus. To strictly restrict cross-province or cross-city mobility in the early wave of pandemic control, various health measures (home isolation, social distancing, travel restriction, and even city lockdown) were implemented by public health authorities [3]. On 23 February 2020, there were 409 newly confirmed patients, 150 new deaths, and 620 new suspected cases in China (China National Health Commission, 2020). Furthermore, on 24 February 2020, the China Bureau for Disease Control and Prevention issued an announcement regarding the scientific and precise prevention and control of COVID-19, which emphasized that farmers’ markets, supermarkets and other necessary living spaces needed to control the number of people and avoid gatherings. Moreover, enterprises and public institutions were suggested to adopt flexible working hours or work-from-home policies, while schools were required to cease in-person operation. Although governments requested that residents practice social distancing and avoid gatherings to reduce the spread of the pandemic, tens of thousands of Chinese tourists ignored the social distancing norm and traveled to Wugong Mountain in Jiangxi Province on 24 February 2020. A similar large gathering was also reported at Huangshan Mountain during the Qingming Festival (i.e., 4–6 April 2020). To control the spread of the epidemic, authorities at Huangshan Mountain issued an emergency notice restricting the number of tourists visiting the site. The mass gathering at scenic spots drew intense criticism on social networking platforms, with messages such as ‘A little stir-crazy’, ‘Doctors died for nothing’, ‘Harming ourselves!’ and ‘So irresponsible’. Although Asian people have collectivistic tendencies and are known to be more inclined to perceive their responsibilities toward others and to follow epidemic prevention guidelines [4], the predictors of their noncompliant behavior need further exploration. The findings on individuals’ noncompliance with health directives and travel during the epidemic could inform strategies by health authorities for population mobility management during pandemics.

Existing research on public compliance with regulations has focused mainly on examining compliance with mask wearing [5], social distancing [6], or home isolation, while compliance with directives on travel and mobility has been less explored. Recent research on compliance with regulations has tried to identify what factors motivate or impede the public’s adherence to health preventative measures. The main influencing factors for positive compliance behavior include risk perception, fear, social responsibility, self-protection, and protection of family members [7,8]. In contrast, noncompliance behavior might occur due to distrust, mixed messages about the epidemic or incapacity to comply [7]. However, the structural relationships between various factors still need further exploration [8]. Furthermore, scholars have emphasized the necessity to explore how different perspectives on health beliefs (e.g., God will protect people [7]) and maladaptive responses [9] lead people to not follow health directives.

To address these gaps, the objectives of this study were to (1) analyze the psychological reason why people do not comply with public health measures and decide to travel; (2) determine the roles of adaptive and maladaptive responses in individuals’ risk-related travel constraint negotiation process; (3) compare the psychology of those who traveled and those who did not travel during epidemics; and (4) examine how obligation influenced people’s tourism decision-making during the pandemic. By revealing tourists’ subtle internal conflicts in the face of health crises, this study aims to contribute to effective policy strategies for population mobility management during a pandemic.

## 2. Theoretical Background and Hypothesis Development

### 2.1. Epidemics and Public Compliance Behavior

Previous epidemiological studies have indicated that the prevention and control of epidemics greatly rely on individuals’ compliance with health directives [10]. For instance, noncompliant infected travelers might result in considerable challenges for the city that received them [11]. For this reason, there is increasing interest in exploring what factors predict individuals’ compliance or noncompliance with preventive measures [8,12].

Overall, existing research has focused on understanding what factors motivate to comply or impede people from complying with health directives [6,10,12,13]. Some factors (e.g., risk perception, fear, trust, self-protection, social responsibility) have been recognized as predictors of public compliance [7]. In contrast, other factors (desire to maintain a normal life, belief in God’s protection, economic concerns, lack of faith in effectiveness, boredom) have been considered variables that impede the acceptance of preventive measures [10,14]. Nevertheless, a dichotomous approach to enhancing or impeding factors might encounter difficulties in explaining public dynamics and complex compliance behavior. For instance, risk perception has been considered a crucial predictor of public compliance [15]. In other words, individuals with stronger health risk perception are more willing to accept protective measures [14]. Nonetheless, risk perception fluctuates over time, and the impact of its two dimensions (i.e., perceived severity, perceived susceptibility) on public compliance might be opposed [8]. Another important predictor of public compliance is trust. Although trust has been repeatedly viewed as vital for promoting public compliance, Wong and Jensen [16] argued that trust might also be a double-edged sword. For example, public trust in government might lead to the underestimation of risk and decreased compliance with health directives [17,18,19]. Further research is needed to investigate how different factors interact and determine compliance behavior [8]. However, previous studies have mainly elucidated what factors directly predict compliance behavior, while the underlying influencing mechanism between motivators and obstacles and the potential mediation process remain underresearched [20].

On the other hand, existing research on public compliance with COVID-19 regulations has mainly focused on examining compliance with mask wearing [5], social distancing [6,21], or home isolation, while compliance with directives on travel and mobility has been less explored. However, the predictors of people’s compliance with directives on travel and mobility might be much more complex [22]. For instance, the desire and benefit of travel could play important roles in influencing travel decision-making [23]. Hence, public decision-making on compliance with directives on travel and mobility during epidemics needs further exploration [24].

### 2.2. Interaction between Travel Motivations and Risk-Related Constraints

To analyze people’s travel behavior during epidemics, it is vital to examine the interaction between motivations and constraints [25]. First, studies on the constraint negotiation process during epidemics have been limited. Initial studies mainly focused on the ‘barriers’ to participation [26], while subsequent research argued that constraints are negotiable and that successful negotiation could lead to participation [27]. Hence, factors that ‘limit people’s participation and enjoyment in leisure activities’ began to be included in definitions of constraints [28], and these definitions can be categorized into three types (i.e., intrapersonal, interpersonal, and structural constraints [29]). The most proximal and powerful type of constraint is intrapersonal constraints (e.g., fear), which depend on individuals’ psychological attributes and affect their preferences and decisions to participate [17,29,30]. The next important type is interpersonal constraints (e.g., lack of company on trips), and the last is structural constraints (e.g., inability to travel long distances) [27]. Risks can be considered intrapersonal constraints that strongly influence the decision to travel [31]; furthermore, they induce or affect interpersonal and structural constraints. Shin [25] found that intrapersonal constraints and structural constraints have a significantly negative effect on intention to travel during a pandemic. Furthermore, previous studies have suggested that a higher perceived risk triggers a greater coping response among people [21,32,33].

**Hypothesis** **1** **(H1).** *Risk-related constraints positively affect coping response*.

**Hypothesis** **2** **(H2).** *Risk-related constraints negatively affect attitude toward traveling*.

**Hypothesis** **3** **(H3).** *Risk-related constraints negatively affect intention to travel*.

To analyze the internal relationship between constraints and other variables (e.g., motivation and negotiation) [34], four alternative models (i.e., the independence, negotiation buffer, constraint effects mitigation, and perceived constraint reduction models) were tested. Among these models, the constraint effects mitigation model proved to be efficient in understanding tourists’ behavior [34,35] and showing that both motivation and constraints trigger negotiation to eliminate the adverse effect of inhibitors [35]. Hence, how individuals negotiate various risk-related constraints and make decisions to travel during epidemics requires further investigation.

**Hypothesis** **4** **(H4).** *Travel motivation positively affects coping responses*.

**Hypothesis** **5** **(H5).** *Travel motivation positively affects attitude toward traveling*.

**Hypothesis** **6** **(H6).** *Travel motivation positively affects intention to travel*.

### 2.3. Coping: Adaptive and Maladaptive Responses

When facing a crisis, human beings may experience fear, distress, and loss of safety and control [36,37]; this is particularly true during the pervasive COVID-19 pandemic, with high morbidity and mortality [38,39]. The dangerous and unpredictable nature of COVID-19, with the necessary isolation measures, affects people’s physical and psychological health [1]. In such situations, to reduce psychological distress evoked by infectious disease, people adopt two types of coping modes [37]: adaptive response [40] and maladaptive response. Adaptive coping is defined as rational problem solving that directly addresses the threat. Seeking and processing pre-travel health information or taking vaccinations prior to travel are the most common adaptive coping responses [9]. In contrast, a maladaptive response can reduce feelings of fear or distress but not directly reduce the threat itself (e.g., wishful thinking, holiday spirit, religious faith, fatalism, and avoidance) [41]. In risky situations, prior experience of overcoming threats without taking any action [42] or confidence in one’s health may encourage people to adopt maladaptive responses [43]. For example, among Australians traveling to Southeast Asia [7], avoidance and holiday spirit were found to be the most common maladaptive responses to health risks (e.g., rabies). Unlike adaptive responses, which encourage protective behavior, maladaptive responses usually weaken preventive strategies [41].

The concepts of adaptive and maladaptive responses have been applied in protective motivation theory (PMT) to understand people’s health protective behavior while traveling. An examination of individuals’ maladaptive perceptions helps clarify why people travel in times of crisis without being concerned about their health [44,45]. The travel decision-making process usually combines intellect and intuition [46]. Individuals rely on unstable and fluid knowledge to ‘feel’, rather than ‘know’, the nature of their hypothetical holidays. Tourists tend to feel that they are unlikely to encounter risks (e.g., be the targets of terrorism) [47]. Furthermore, they attempt to implement strategies to rationalize their travel behavior in risky situations [48]. When facing crises, tourists exhibit coping responses that increase their feeling of control over the risky situation, which, in turn, influences their intention to travel [49].

**Hypothesis** **7** **(H7).** *Coping responses positively affect attitudes toward traveling*.

**Hypothesis** **8** **(H8).** *Coping responses positively affect intention to travel*.

### 2.4. Perceived Obligation and Attitude/Intention

As suggested by many scholars, the reaction of prospective tourists to infectious diseases may be related to cultural tendencies [4,50]; specifically in the context of China, this reaction may be related to collectivist tendencies based on Confucian philosophy. When confronted with infectious diseases, collectivists are more connected (physically and mentally) to group members than individualists, are more able to visibly perceive differences within and outside the group [4,51] and are therefore more wary of exposure to the unknown (e.g., outsiders and viruses) [4], and are more likely to consciously take responsibility for epidemic prevention out of fear of transmission to in-group members (e.g., family, friends, colleagues, etc.) and comply with precautionary measures, thus exhibiting a higher level of perceived obligation, self-consciousness, and responsibility [52]. Many scholars have emphasized the need to study the impact of perceived obligations on tourism decisions during and after a crisis [53], because when the epidemic subsides and some scenic spots reopen, shared negative experiences and emotions make collectivists more inclined to rely on each other [54,55,56], perceive themselves as existing in a collective, and prefer not to travel to avoid posing a threat to the collective.

**Hypothesis** **9** **(H9).** *Perceived obligation negatively affects attitude toward traveling*.

**Hypothesis** **10** **(H10).** *Perceived obligation negatively affects intention to travel*.

In addition, attitude was also incorporated as a construct to better understand its mediating effect on the relationships between travel motivation and intention [57] and risk-related constraints and intention [25].

**Hypothesis** **11** **(H11).** *Attitude toward traveling positively affects intention to travel*.

**Hypothesis** **12** **(H12).** *Attitude mediates the relationship between risk-related constraints and intention to travel*.

**Hypothesis** **13** **(H13).** *Attitude mediates the relationship between motivation and intention to travel*.

**Hypothesis** **14** **(H14).** *Attitude mediates the relationship between perceived obligation and intention to travel*.

**Figure 1:**
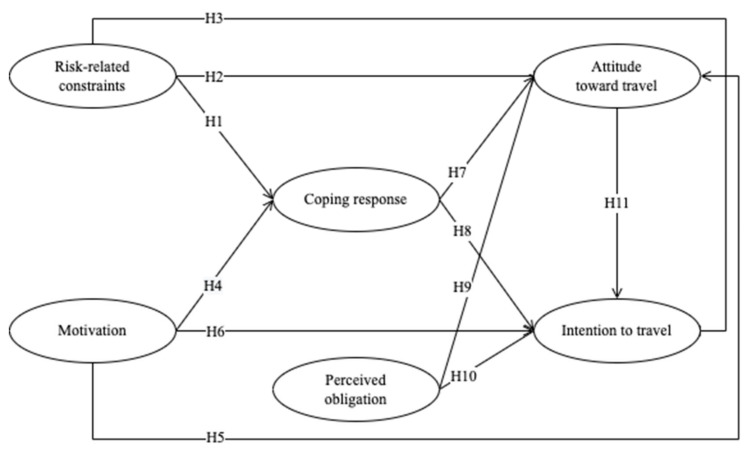
Conceptual framework.

To address this gap, this study aims to clarify the underlying psychological mechanism of individuals’ noncompliance with directives on travel and mobility during the pandemic (Figure 1).

## 3. Methods

### 3.1. Measurement Scale

According to the procedures proposed by Churchill [58], a measurement was developed in accordance with the relevant literature (Appendix A) and online comments collected from two social media platforms (i.e., Sina Microblog (Beijing, China) and Zhihu (Beijing, China)). After conducting a pilot survey on 100 individuals, the cross-loading items were deleted. Furthermore, risk-related constraints were measured using 13 items [31,35,40,59]. To capture the participants’ motives for outdoor leisure or travel, items (e.g., relaxation, enjoying beautiful natural scenery) were developed based on the review by Zhang [60] on travel motivation. Subsequently, adaptive response was measured using statements such as seeking information and actions to prevent viral infection, whereas maladaptive perception was measured using six items. Subsequently, questions on perceived obligation [61] and participants’ attitude and intention to travel [57] were designed. Amidst the epidemic crisis, the demand for local leisure and vacation has also increased. Hence, the intention constraint included local leisure in urban parks. These questions were rated on a five-point Likert scale, ranging from 1, ‘totally disagree’, to 5, ‘totally agree’.

### 3.2. Setting and Sampling

The sample comprised Chinese residents who had experienced the outbreak of COVID-19 and quarantine restrictions since 23 January 2020. Due to the restrictions of isolation, only online surveys were feasible. First, a pilot study with 100 respondents was conducted to test the applicability and appropriateness of the measurements. Then, the formal survey employed a quota sampling procedure to ensure wide geographical dispersal [53] across different provinces with different infection rates in China (e.g., Hubei) by cooperating with a professional survey company (www.wenjuanxing.com (accessed on 8 May 2020)). The first survey was conducted from 28 February to 10 March 2020, and the second was conducted from 1 May to 8 May 2020, with each survey recording 1000 responses. The participants were told that the survey was anonymous and confidential. After finishing the questionnaire, they were provided ¥2 (approximately $0.31) for compensation. After the responses that were finished in less than three minutes and those with suspicious response patterns were excluded, the final number of participant responses was 1473 (i.e., 700 for round 1 and 773 for round 2). Female respondents accounted for 59% and 58.6% of the round 1 and round 2 samples, respectively. Furthermore, in rounds 1 and 2, 51.9% and 42.9% of the respondents in rounds 1 and 2 had received a college education, 17.6% and 20.6% of respondents were aged 30–39 years, and respondents aged 40–59 years accounted for 16.8% and 23.4%, respectively. The two samples covered all provinces and regions of mainland China. Furthermore, the respondents’ occupations were diverse, including corporate employees, government workers, farmers, migrant workers, medical workers, and retirees. Since 25 February 2020, 3.9% and 24.6% of the respondents in the first- and second-round samples, respectively, have traveled to different destinations.

### 3.3. Data Analysis Methods

First, an exploratory factor analysis (EFA) was conducted to extract the constructs of travel motivation, constraints, coping responses, attitude, and intention. Next, an independent *t* test was performed to compare motivation and constraints between the respondents in rounds 1 and 2. Finally, the hypothetical relationships were examined using Amos 21.0.

## 4. Results

### 4.1. Travel Motivations and Risk-Related Constraints

To identify the dimensions of travel motivation, an EFA was performed. After the cross-loading factors (i.e., moti5, pull5, and moti9) were excluded, four factors were obtained (Table 1). The first factor, labeled ‘relaxation and proximity to nature’, highlights the necessity of outdoor leisure and travel. The second factor is related to the pull motivation adopted by tourism destinations to attract tourists by eliminating any risks. The third factor, ‘ordinary motivation’, reflects tourists’ common motives. The fourth factor, ‘peak shifting motivation’, is related to traveling in scenic spots with limited visitors. The Cronbach’s alpha values of the four factors were between 0.826 and 0.923, which suggests satisfactory reliability (Table 1). 

To identify the construction of risk-related constraints, an EFA was conducted using varimax rotation. Owing to low factor loadings, struct1 (i.e., lack of time) and struct5 (i.e., lack of money) were deleted. Then, three factors were obtained that explained more than 69% of the total variance for rounds 1 and 2. The Cronbach’s alpha values of the three factors were at the threshold of 0.70 [62], which suggests satisfactory reliability. All three types of constraints in round 1 were significantly higher than those in round 2 (Table 1).

### 4.2. Coping, Obligation, Attitude, and Intention to Travel

Before the EFA, perceived obligation, coping response, attitude and intention were tested for validity using the KMO and Bartlett’s spherical approximate chi-square test. The KMO values for each variable ranged from 0.71 to 0.87, and the chi-square test values ranged from 1280.3 to 2724.7 for both rounds. Moreover, the significant probability values for the latter were well below 0.05 (*p* < 0. 000). Therefore, the results reject the hypothesis that the correlation matrix is not a unit matrix, so the data are suitable for EFA.

According to the criterion [63] that only items with factor loadings >0.4 in absolute value are included in the constructs, four question items (Moti5, Struct1, Struct5 and Moti9) were excluded from this study. Among the coping responses, three factors were obtained by EFA in the first and second rounds, explaining more than 71% and 70% of the variance, respectively (Table 2). The first factor, “holiday spirit and denial”, reflects individuals’ underestimation of the possible health risks associated with travel. The second factor, “religious faith and wish”, highlights people’s belief that spirits will bless them during travel. The third factor, “adaptive response”, refers to people’s responses in terms of seeking information and following social distance norms; factor loadings range from 0.58 to 0.88 (Table 2).

A principal component analysis was performed separately for the three factors of perceived obligation, attitude, and intention. The analysis results revealed that the factors had a factorial structure that was near or exceeded 70% of the total variance (Table 2). Perceived obligation included three items. In addition, attitude included three measurements—suitable, pleasant, and safe—whereas intention included five measurements (e.g., visit natural scenic spots).

### 4.3. Comparison of Those Who Traveled and Those Who Did Not

One-way ANOVA of travelers and nontravelers in both rounds of the survey revealed that both those who traveled and those who did not travel agreed that they had a high social obligation to comply with the preventative measures. At the same time, travel motivation, maladaptive response, attitude and intention were all higher for travelers than for nontravelers in both rounds of the study, which indicates that higher travel motivation and a stronger maladaptive response of holiday spirit and denial encouraged noncompliance with health directives and travel amidst threat (Table 3).

### 4.4. Dynamic Travel Decision-Making during the Pandemic

#### 4.4.1. Measurement Models

The measurement model for travel motivation, constraints, coping response, attitude, and intention was tested using CFA in Amos 21.0 software. The composite reliability (CR) for all constructs [64] exceeded the general criterion of 0.60, which demonstrates the good internal consistency of the constructs. The average variance extracted (AVE) [65] for all the constructs was above the threshold value of 0.5, which indicates that the observed items could explain the latent variables well.

#### 4.4.2. Hypothesis Testing

The structural equation model (SEM) was estimated using the maximum likelihood estimation method. The results indicated a satisfactory fit for the model (round 1 (N = 700): χ^2^/df = 2.802, *p* < 0.000, RMSEA = 0.051, NFI = 0.889, CFI = 0.925, TLI = 0.919, IFI = 0.925, PGFI = 0.753; round 2 (N = 773): χ^2^/df = 2.922, *p* < 0.000, RMSEA = 0.050, NFI = 0.877, CFI = 0.915, TLI = 0.908, IFI = 0.915, PGFI = 0.759). Table 4 shows the path coefficient between the constructs.

**Figure 2:**
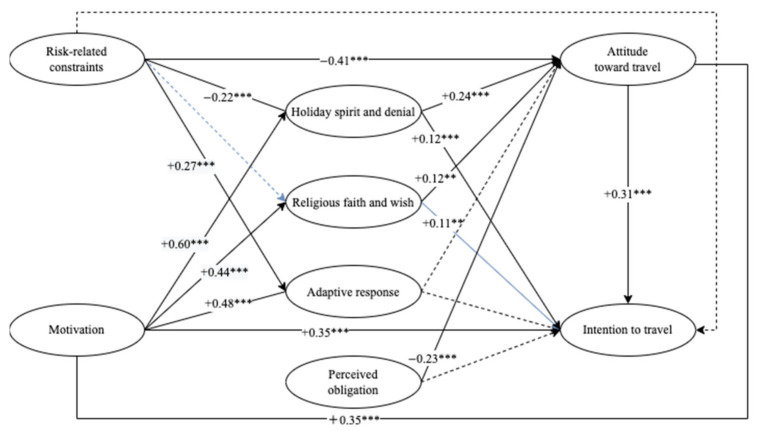
Results of hypothesis model (Round 1). Note: ** *p* < 0.05, *** *p* < 0.01.

**Figure 3:**
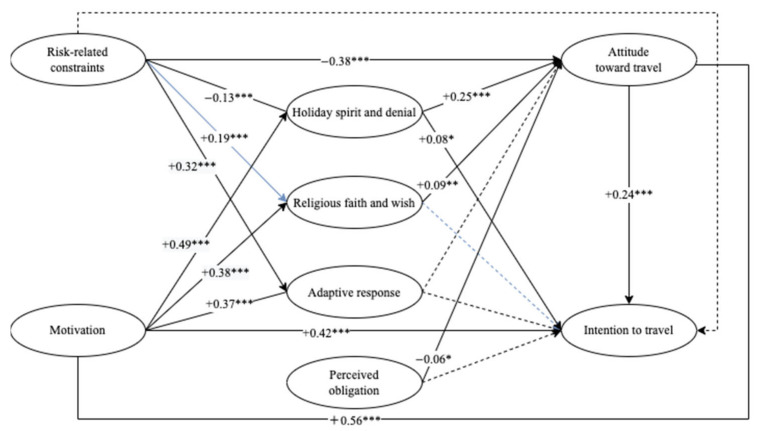
Results of hypothesis model (Round 2). Note: * *p* < 0.1, ** *p* < 0.05, *** *p* < 0.01.

The results partially supported Hypothesis 1, which proposed that stronger constraints trigger more coping responses. When individuals encounter more intense constraints, they adopt both adaptive and maladaptive responses. The second hypothesis (H2) on the negative effect of constraints on attitude (β = −0.31, −0.36) was supported, whereas the negative effect of constraints on intention was not significant (H3). On the other hand, travel motivation could activate coping responses (H4) and positively influence attitude (H5) and behavioral intention (H6). The seventh hypothesis (H7), which proposed that coping responses have a positive effect on attitude, was partially supported. Adaptive responses could not significantly promote a positive attitude toward traveling during epidemics. The effects of coping responses on intention to travel (H8) were partially supported. Perceived obligation had a negative effect on attitudes toward traveling (β = −0.23, −0.06) (H9), while its effect on intentions to travel was not significant (H10). The eleventh hypothesis (H11), which proposed that attitude significantly influences intention to travel, was supported (β = 0.29, 0.24) (Figure 2 and Figure 3).

#### 4.4.3. Mediating Effects

To explore the indirect effects of attitude, the widely used approach of bootstrapping [66] and 2000-sampling tests [67,68] were conducted using Amos 21.0 software. The results indicated that the indirect mediating effect of attitude on the relationship between constraint and intention was significant (H12) in both round 1 (β = −0.2410 *, 95% confidence interval (CI) = −0.391 to −0.099) and round 2 (β = −0.111 *, 95% CI = −0.181 to −0.048). On the other hand, travel motivation affected intention not only indirectly through attitude (β = 0.406 *, 95% CI = 0.294 to 0.561) (H13) but also directly. The partial mediating effect of attitude on this relationship was verified for round 2 as well. In addition, perceived obligation influenced intention indirectly via attitude in round 1 (β = −0.090 **, 95% CI = −0.143 to −0.048), while the indirect effect of attitude was not significant in round 2 (H14) (Table 5).

## 5. Discussion

Human mobility greatly increases the risk of epidemic transmission. Some scholars have found that the reaction of prospective tourists to infectious diseases may be related to cultural tendencies [4,49]. Collectivists are more inclined to perceive their responsibility to others and comply with epidemic guidelines, but predictors of their noncompliance need to be further explored. Therefore, to reveal the subtle internal conflicts of tourists in the face of health crises, this study explored the psychological mechanisms by which individuals do not comply with public health directives and choose to travel amidst pandemic threats. The results of this study will contribute to policies for managing population movements during pandemics.

First, empirical evidence for hypotheses H1~H6 suggests that the relative strength of the motivating and impeding factors determines a person’s compliance with directives on travel and mobility. Specifically, on the one hand, the hypotheses of H1, H2 and H3 are consistent with previous studies, and risk-related variables (fear and depression [69]) promote compliance behavior [15,19,70]. On the other hand, empirical evidence for hypotheses H4, H5 and H6 reveals the motivation of potential travelers to travel, indicating that the desire for ‘relaxation and proximity to nature’ emerged as the most important motivating factor that encouraged travel amidst the pandemic. This was consistent with Kaim’s study [10], which suggested that ‘a desire to maintain a normal life routine’ leads to noncompliance behavior. The strict ‘self-quarantine’ campaign restrained people’s leisure demand, thus triggering a strong desire for outdoor recreation in the later stages of the epidemic. Moreover, some people believed that their participation in leisure activities and the optimistic experience of seeking happiness could increase their resilience [71].

If there was a reduction in the threat, risk-related constraints significantly decreased, whereas travel motivation increased. The change in the relative strength of motivators and obstacles could explain individuals’ more or less strict compliance.

Second, the evidence empirically supports H7 and H8 that coping responses positively affect individuals’ attitudes toward travel and intention to travel, revealing the vital role of maladaptive responses (e.g., wishful thinking, denial, fatalism) in encouraging noncompliance with directives on mobility. Although maladaptive response is a predictor of individuals’ health protective behavior, this concept has largely been neglected in tourism crisis studies [9]. During the unprecedented COVID-19 pandemic, people have tended to adopt maladaptive responses (e.g., holiday spirit or following one’s religious faith) to reduce their fear or distress [41] and increase their sense of control [9]. By comparing those who traveled and those who did not, the results showed that the ‘holiday spirit and denial’ of those who traveled was significantly higher. Maladaptive responses lead to the underestimation of risk and noncompliance behavior through mass gathering at scenic spots. This is supported by Wang’s study [7], which found that respondents who believed in their immune system and trusted in God were reluctant to comply with health measures. Kaim [10] also revealed that individuals’ beliefs that God blessed them acted as an obstacle to complying with health measures. The current study contributes to knowledge of how maladaptive responses interact with travel motivation and risk-related constraints to influence compliance behavior.

Third, the empirical support for H9 and H10 in this study helps expand the understanding of how obligations directly or indirectly affect public compliance behavior [56,70]. The results demonstrated that most respondents strongly agreed that they had a responsibility toward others for epidemic prevention. As stated by previous research, social responsibility seems to have a direct impact on compliance behavior [7]. However, whether other variables mediate the relationship between obligation and compliance behavior remains unclear. Furthermore, the present study’s finding that the effect of representative obligations on compliance behavior for hypothesis H14 occurs exclusively through attitudes as mediators [55] extends previous research by suggesting that obligations indirectly influence compliance with travel and mobility instructions through the mediating role of attitudes. Interestingly, both those who traveled and those who did not travel agreed with their obligation to take preventive measures; however, they exhibited the opposite behavior. This might be because those who traveled thought that the scenic areas were empty so that there was little possibility of mass gathering and a low risk of spreading the virus. In this sense, they did not seem to perceive their behavior as disobeying the heath measures. In other words, they might strictly comply with the measures in farmers’ markets but not necessarily in outdoor open spaces. It has been suggested that people might perceive the recommended preventive behavior differently in different settings [7].

## 6. Conclusions

As the global pandemic has rapidly spread across geographical boundaries, human travel behavior has had a significant effect on how the epidemic developed. This study sheds new light on the promotion of public compliance to directives on travel and mobility by exploring the underlying influencing mechanism between motivators and obstacles, as well as the potential mediation process. The findings reveal that the relative strength of the effect of motivating and impeding factors and mediators determines whether individuals comply with directives on travel and mobility. Moreover, maladaptive responses significantly encourage travel amidst threat. Furthermore, the impact of obligation on compliance behavior occurs solely via attitude as a mediator [56]. However, this study is not without limitations. First, since tourists with different cultural backgrounds [2] might exhibit different behaviors, future research could compare compliance decision-making processes in different cultural contexts to generalize the results. Second, since the pandemic continues to be an active threat, a comparative study of people’s internal conflicts at multiple time points [40] might yield more relevant discoveries.

The results provide several implications for the management of human compliance with directives on travel and mobility during epidemics.

First, since public compliance behavior depends on the stage of the epidemic and dynamic psychological processes, destination management organizations and governments should use different approaches to provide sufficient information to meet people’s need for risk understanding [72,73,74], especially during the current phase of regular epidemic prevention and control, to properly remind travelers of their responsible behavior and prevent paralysis. On the one hand, the grading, zoning, and real-time disclosure of health prevention and travel and mobility control policies are essential, especially to raise awareness among travelers of risks that they think are unlikely to occur [75]; on the other hand, the review of information disseminated by social media in public places should be strengthened to prevent exaggerated safety levels in tourist attractions and exaggerated risk levels in the news and social media [72,73,74] and to ensure the accuracy and validity of mass communication information [76]. This could help prevent individuals from feeling uncertain about their responsibilities and help them avoid unknowingly traveling to risk-generating regions.

Second, many people believe that God can protect them from the epidemic, thus neglecting or trivializing personal health protective behaviors and creating barriers to compliance with health measures, i.e., an individual’s prediction of the likelihood of infection can directly influence personal protective behaviors. It is therefore necessary to consider other ways, such as cultural, religious and political, to promote voluntary self-protective behavior during epidemics. For example, cultural and religious authorities and the government can use their status to disseminate information about the epidemic and advocate or ask people not to travel during an epidemic or infection, which can improve people’s perception of the risk of infection.

Finally, although we have now entered the normalized phase of epidemic prevention and control, the epidemic has still rebounded from time to time in local areas. With the inability to travel, safer and more convenient virtual tourism has encountered development opportunities [75,77,78]. In this regard, tourism enterprises, scenic spots, and practitioners should actively expand their online businesses to provide residents with abundant virtual tourism products (e.g., healthy and green themes) that not only satisfy people’s desire to travel to a certain extent, improve residents’ mental health [79], and bring significant emotional recovery and stress relief benefits to individuals but also help maintain user stickiness with tourists and provide a foundation for actual travel by tourists after the epidemic subsides [80,81,82].

However, this study is not without limitations. First, since tourists with different cultural backgrounds [2] might exhibit different behaviors, future research could compare compliance decision-making processes in different cultural contexts to generalize the results. Second, since the pandemic continues to be an active threat, a comparative study of people’s internal conflicts at multiple time points [40] might yield more relevant discoveries.

## Figures and Tables

**Table 1:** Factor analyses on travel motivation and constraints items and the result of *t* test.

Factor and Item	EFA (Round1 N = 700)				EFA (Round2 N = 773)				*t*	Probability
	Mean	Loading	Eigenvalue	Variance Explained (%)	Mean	Loading	Eigenvalue	Variance Explained (%)		
♦ **Relaxation and proximity to nature (α = 0.92/0.87)**	**3.93**		**7.66**	**51.06**	**4.03**		**6.94**	**46.27**	**−2.35**	**0.019**
Moti1 relax in a beautiful location	3.93	0.85			4.09	0.78				
Moti2 visit scenic spots to exercise my muscles and bones	3.86	0.84			3.95	0.78				
Moti3 enjoy the beautiful natural scenery	3.95	0.83			4.11	0.84				
Moti4 visit sunny places and relieve my anxiety regarding the epidemic	3.98	0.83			3.98	0.76				
♦ **Pull motives (α = 0.91/0.89)**	**3.40**		**1.87**	**12.43**	**3.68**		**1.75**	**11.63**	**−6.78**	**0.000**
Pull1 The online booking for ticket reduces risks.	3.61	0.74			3.85	0.74				
Pull2 The scenic spot is equipped with hardware and software for epidemic prevention and control, which dispels my worries.	3.20	0.86			3.51	0.83				
Pull3 The scenic spot uses big data to fully understand the health status of tourists.	3.26	0.87			3.60	0.88				
Pull4 The scenic spot implements time-based and decentralized tours, which are relatively safe.	3.49	0.84			3.78	0.83				
♦ **Ordinary motives (α = 0.88/0.83)**	**3.40**		**1.24**	**8.25**	**3.59**		**1.21**	**8.05**	**−4.42**	**0.000**
Moti10 seek novelty	2.77	0.62			2.94	0.67				
Moti11 increase knowledge through travel	3.43	0.84			3.58	0.82				
Moti12 experience a different culture and life	3.66	0.83			3.86	0.79				
Moti13 spend a happy time with family/friends	3.74	0.71			3.99	0.60				
♦ **Peak shifting motives (α = 0.87/0.85)**	**3.14**		**1.08**	**7.17**	**3.43**		**1.11**	**7.40**	**−5.84**	**0.000**
Moti6 enjoy the beautiful scenery alone	3.15	0.83			3.41	0.81				
Moti7 easier to find an angle to avoid the crowd when taking pictures	3.24	0.82			3.43	0.86				
Moti8 take advantage of the recent free time to travel	3.05	0.73			3.47	0.67				
Round1: KMO = 0.92, Bartlett’s test of sphericity: χ^2^ = 8151.98, *p* < 0.000, α = 0.93, 78.92%; KMO = 0.91, Bartlett’s test of sphericity: χ^2^ = 7011.28, *p* < 0.000, α = 0.92, 73.35%;										
♦ **Intrapersonal constraints (EFA α = 0.85/0.85)**	**3.83**		**1.47**	**13.40**	**3.59**		**5.23**	**47.57**	**6.29**	**0.000**
Intra1 I worry about contracting COVID-19 while travelling in scenic spots.	3.77	0.77			3.57	0.85				
Intra2 I am worried about contracting COVID-19 while using transportation facilities.	3.98	0.78			3.72	0.84				
Intra3 I am afraid of COVID-19 and worried about becoming infected.	3.75	0.72			3.46	0.62				
Intra4 I don’t like taking risks; I cherish life.	3.80	0.83			3.60	0.82				
♦ **Interpersonal constraints (EFA α = 0.79/0.72)**	**3.71**		**1.16**	**10.52**	**3.33**		**1.00**	**9.07**	**9.75**	**0.000**
Inter1 hard to find a companion	3.62	0.82			3.36	0.76				
Inter2 My family and friends disagree with my intention to travel now.	3.94	0.74			3.49	0.75				
Inter3 I am worried about being criticized by others for travelling now.	3.58	0.80			3.14	0.72				
♦ **Structural constraints (EFA α = 0.84/0.85)**	**4.00**		**5.02**	**45.69**	**3.69**		**1.38**	**12.57**	**8.07**	**0.000**
Struct2 Hotels, restaurants, etc. are risky.	3.82	0.67			3.67	0.75				
Struct3 The external environment is too complicated.	4.01	0.85			3.65	0.83				
Struct4 encounter many difficulties and inconveniences	4.07	0.85			3.71	0.77				
Struct6 I can travel anytime, and I don’t have to do it now.	4.02	0.71			3.72	0.70				
Round1: KMO = 0.87, Bartlett’s test of sphericity: χ^2^ =3686.50, *p* < 0.000, α = 0.93, 69.59%; Round2: KMO = 0.88, Bartlett’s test of sphericity: χ^2^ =4310.292, *p* < 0.000, α = 0.92, 69.21%										

**Table 2:** Results of the factor analyses on perceived obligation, coping response, attitude and intention items.

Factor and Items		EFA (Round1 N = 700)			EFA (Round2 N = 773)			
	Mean	Loading	Eigenvalue	Variance Explained (%)	Mean	Loading	Eigenvalue	Variance Explained (%)
♦ **Perceived obligation (EFA α = 0.79/0.92)**	**4.39**		**2.60**	**86.72**	**4.19**		**2.48**	**82.67**
Ob1 I feel obligated to stay at home to support epidemic prevention and control.	4.32	0.91			4.10	0.90		
Ob2 I feel obligated to ensure adherence to the restrictions of home isolation so as to respect others.	4.41	0.95			4.23	0.93		
Ob3 I feel obligated to main vigilance in the critical period of epidemic prevention.	4.44	0.93			4.25	0.90		
Round1: KMO = 0.74, Bartlett’s test of sphericity: χ^2^ = 1650.92, *p* < 0.000; Round2: KMO = 0.74, Bartlett’s test of sphericity: χ^2^ = 1394.93, *p* < 0.000								
♦ **Holiday spirit and denial (EFA α = 0.79/0.79)**	**2.84**		**3.22**	**40.23**	**3.03**		**2.99**	**37.35**
Cop1 I am always in good health and have a strong immune system; so, I will be fine when traveling.	3.04	0.79			3.20	0.83		
Cop2 I wear a mask and wash my hands in time; so, I don’t have to worry about infection.	2.81	0.87			3.04	0.86		
Cop3 I try not to think too much about the health risks every time I travel.	2.67	0.75			2.84	0.74		
♦ **Religious faith and wish (EFA α = 0.66/0.68)**	**3.20**		**1.37**	**17.12**	**2.90**		**1.49**	**18.63**
Cop6 I believe that any health issues I might encounter during travelling will be miraculously cured in the future.	3.51	0.76			3.42	0.68		
Cop7 God will protect me from the virus.	2.90	0.82			2.79	0.85		
Cop8 If it’s your destiny to get infected during your trip, there is little you can do.	2.39	0.58			2.50	0.74		
♦ **Adaptive response (EFA α = 0.74/0.74)**	**3.65**		**1.12**	**14.04**	**3.73**		**1.17**	**14.56**
Cop4 Wait in line at least 1.5 m apart from another person and try to avoid crowded areas.	3.59	0.84			3.69	0.84		
Cop5 Seek information on the progress of epidemic prevention and control.	3.71	0.85			3.77	0.88		
Round1: KMO = 0.73, Bartlett’s test of sphericity: χ^2^ = 1787.81, *p* < 0.000,α = 0.78, 71.38%; Round2: KMO = 0.71, Bartlett’s test of sphericity: χ^2^ = 1790.70, *p* < 0.000, α = 0.75, 70.55%								
♦ **Attitude (EFA α = 0.90/0.90)**	**2.22**		**2.49**	**82.87**	**2.93**		**2.48**	**82.81**
Atti1 Travelling now is suitable.	2.11	0.93			2.83	0.92		
Atti2 Travelling now is pleasant.	2.49	0.90			3.14	0.88		
Atti3 Travelling now is safe.	2.07	0.91			2.83	0.92		
Round1: KMO = 0.74, Bartlett’s test of sphericity: χ^2^ =1280.30, *p* < 0.000; Round2: KMO = 0.74, Bartlett’s test of sphericity: χ^2^ =1432.42, *p* < 0.000								
♦ **Intention (EFA α = 0.93/0.89)**	**3.00**		**3.86**	**77.15**	**3.56**		**3.46**	**69.14**
Intent1 I plan to go to an urban park.	3.26	0.84			3.74	0.76		
Intent2 I plan to taste nearby cuisine.	2.95	0.85			3.53	0.77		
Intent3 I desire to visit short-distance tourist attractions.	2.86	0.92			3.48	0.88		
Intent4 I desire to travel to low-risk destinations.	2.92	0.90			3.47	0.86		
Intent5 I intend to visit natural scenic spots.	3.02	0.89			3.58	0.87		
Round1: KMO = 0.87, Bartlett’s test of sphericity: χ^2^ = 2724.70, *p* < 0.000; Round2: KMO = 0.85, Bartlett’s test of sphericity: χ^2^ = 2209.42, *p* < 0.000								

**Table 3:** Comparison between those who travelled and who didn’t.

Constructs	Round1 (N = 700)				Round2 (N = 773)			
	Travelled (*n* = 27)	Didn’t Travel (*n* = 673)	Overall Mean	One-Way ANOVA	Travelled (*n* = 190)	Didn’t Travel (*n* = 583)	Overall Mean	One-Way ANOVA
Intrapersonal	3.60	3.84	3.83	2.810	3.45	3.63	3.59	8.017
Interpersonal	3.78	3.71	3.71	0.195	3.11	3.40	3.33	21.607
Structural	3.81	3.98	3.98	1.728	3.46	3.76	3.69	25.425
Relaxation	4.43	3.91	3.93	8.204	4.25	3.96	4.03	21.389
Pull motives	4.04	3.36	3.39	15.612	3.73	3.34	3.43	22.548
Ordinary motives	3.85	3.38	3.40	7.214	3.78	3.53	3.60	15.132
Peak shifting motives	3.77	3.12	3.14	11.169	3.73	3.34	3.43	29.035
Perceived obligation	4.35	4.39	4.39	0.142	4.12	4.22	4.19	2.713
Holiday spirit and denial	3.44	2.82	2.84	14.678	3.24	2.96	3.03	19.016
Religious faith and wish	3.54	3.19	3.20	3.483	2.98	2.87	2.90	1.858
Adaptive response	4.35	4.39	4.39	0.142	3.73	3.73	3.73	0.005
Attitude	3.04	2.19	2.22	24.329	3.34	2.80	2.93	56.096
Intention	3.91	2.97	3.00	27.466	3.88	3.46	3.56	48.613

**Table 4:** Summary of hypotheses test results.

Structural Relations	Standardized Coefficient (b)		*t*-Value		*p*		Contrast	
	Round1	Round2	Round1	Round2	Round1	Round2	Round1	Round2
H1 constraints → holiday spirit and denial	−0.22	−0.13	−5.16	−3.25	***	***	**√**	**√**
Constraints → religious faith and wish	0.07	0.20	1.40	3.60	0.16	***	×	**√**
constraints → adaptive response	0.28	0.32	5.67	6.67	***	***	**√**	**√**
H2 constraints → attitude	−0.31	−0.36	−6.94	−8.88	***	***	**√**	**√**
H3 constraints → intention	−0.05	0.05	−1.17	1.15	0.24	0.25	×	×
H4 motivation → holiday spirit and denial	0.60	0.49	11.62	10.15	***	***	**√**	**√**
motivation → religious	0.44	0.38	7.73	5.76	***	***	**√**	**√**
motivation → adaptive response	0.48	0.37	9.10	7.50	***	***	**√**	**√**
H5 motivation → attitude	0.38	0.44	5.78	8.52	***	***	**√**	**√**
H6 motivation → intention	0.36	0.56	5.61	8.79	***	***	**√**	**√**
H7 holiday spirit and denial → attitude	0.21	0.24	4.37	5.99	***	***	**√**	**√**
religious faith and wish → attitude	0.09	0.09	1.89	2.12	0.06	0.03	**√**	**√**
adaptive response → attitude	0.06	−0.03	1.24	−0.72	0.21	0.47	×	×
H8 holiday spirit and denial → intention	0.12	0.08	2.69	2.03	***	0.04	**√**	**√**
religious faith and wish → intention	0.11	0.01	2.55	0.11	0.01	0.91	√	×
adaptive response → intention	0.07	0.00	1.62	0.08	0.11	0.94	×	×
H9 perceived obligation → attitude	−0.23	−0.06	−7.08	−1.85	***	0.06	**√**	√
H10 perceived obligation → intention	−0.03	0.02	−0.96	0.66	0.34	0.51	×	×
H11 attitude → intention	0.29	0.24	6.06	4.52	***	***	√	√

Note: *** *p* < 0.01.

**Table 5:** Bootstrapping methodology for mediating effect.

Mediating Effects	Total Effects	Direct Effect (CI)	Indirect Effects (CI)	Mediation Hypotheses
**Round 1 (N = 700)**				
constraint-attitude-intention	−0.346 *	−0.105 (−0.318 to 0.068)	−0.241 * (−0.391 to −0. 099)	full mediation
motivation-attitude-intention	0.841 *	0.435 * (0.267 to 0.636)	0.406 * (0.294 to 0.561)	partial mediation
obligation-attitude-intention	−0.129 **	−0.040 (−0.134 to 0.049)	−0.090 ** (−0.143 to −0.048)	full mediation
**Round 2 (N = 773)**				
constraint-attitude-intention	−0.055 **	0.056 (−0.046 to 0.158)	−0.111 ** (−0.181 to −0.048)	full mediation
motivation-attitude-intention	0.702 *	0.535* (0.412 to 0.701)	0.167 ** (0.094 to 0.246)	partial mediation
obligation-attitude-intention	0.005	0.017(−0.054 to 0.086)	−0.011 (−0.028 to 0.003)	Non-significant

Note: * *p* < 0.1; ** *p* < 0.05; bootstrap confidence in parentheses, CI = confidence interval.

## Data Availability

All data used during the study were provided by a third party Sojump (www.wjx.cn, accessed on 23 January 2020)—the largest professional survey company in China. The raw/processed data required to reproduce these findings cannot be shared at this time as the data also forms part of an ongoing study.

## Appendix A

**Table A1:** List of Questionnaire Items.

Factor	Item	Reference
Relaxation and proximity to nature	relax in a beautiful location	Zhang et al., 2021 [60]
	visit scenic spots to exercise my muscles and bones	
	enjoy the beautiful natural scenery	
	visit sunny places and relieve my anxiety regarding the epidemic	
Pull motives	The online booking for ticket reduces risks.	
	The scenic spot is equipped with hardware and software for epidemic prevention and control, which dispels my worries.	
	The scenic spot uses big data to fully understand the health status of tourists.	
	The scenic spot implements time-based and decentralized tours, which are relatively safe.	
Ordinary motives	seek novelty	
	increase knowledge through travel	
	experience a different culture and life	
	spend a happy time with family/friends	
Peak shifting motives	enjoy the beautiful scenery alone	
	easier to find an angle to avoid the crowd when taking pictures	
	take advantage of the recent free time to travel	
Intrapersonal constraints	I worry about contracting COVID-19 while travelling in scenic spots.	Cahyanto et al., 2016 [40] Chen et al., 2013 [31] Hung and Petrick, 2012 [35] Nyaupane and Andereck, 2008 [59]
	I am worried about contracting COVID-19 while using transportation facilities.	
	I am afraid of COVID-19 and worried about becoming infected.	
	I don’t like taking risks; I cherish life.	
Interpersonal constraints	hard to find a companion	
	My family and friends disagree with my intention to travel now.	
	I am worried about being criticized by others for travelling now.	
Structural constraints	Hotels, restaurants, etc. are risky.	
	The external environment is too complicated.	
	encounter many difficulties and inconveniences	
	I can travel anytime, and I don’t have to do it now.	
Perceived obligation	I feel obligated to stay at home to support epidemic prevention and control.	Hartanto et al., 2020 [61]
	I feel obligated to ensure adherence to the restrictions of home isolation so as to respect others.	
	I feel obligated to main vigilance in the critical period of epidemic prevention.	
Holiday spirit and denial	I am always in good health and have a strong immune system; so, I will be fine when traveling.	Wang et al., 2019 [9]
	I wear a mask and wash my hands in time; so, I don’t have to worry about infection.	
	I try not to think too much about the health risks every time I travel.	
Religious faith and wish	I believe that any health issues I might encounter during travelling will be miraculously cured in the future.	
	God will protect me from the virus.	
	If it’s your destiny to get infected during your trip, there is little you can do.	
Adaptive response	Wait in line at least 1.5 m apart from another person and try to avoid crowded areas.	
	Seek information on the progress of epidemic prevention and control.	
Attitude	Travelling now is suitable.	Hung and Hsu, 2009 [57]
	Travelling now is pleasant.	
	Travelling now is safe.	
Intention	I plan to go to an urban park.	Hung and Hsu, 2009 [57]
	I plan to taste nearby cuisine.	
	I desire to visit short-distance tourist attractions.	
	I desire to travel to low-risk destinations.	
	I intend to visit natural scenic spots.	
